# Supplementary material for: Structure mapping of dengue and Zika viruses reveals functional long-range interactions
Source: Nat Commun. 2019 Mar 29;10:1408. doi: 10.1038/s41467-019-09391-8 (PMC6441010; doi:10.1038/s41467-019-09391-8)
Supplement: Supplementary file 1 — Supplementary Information [file 41467_2019_9391_MOESM1_ESM.pdf]

Supplementary Figure 1

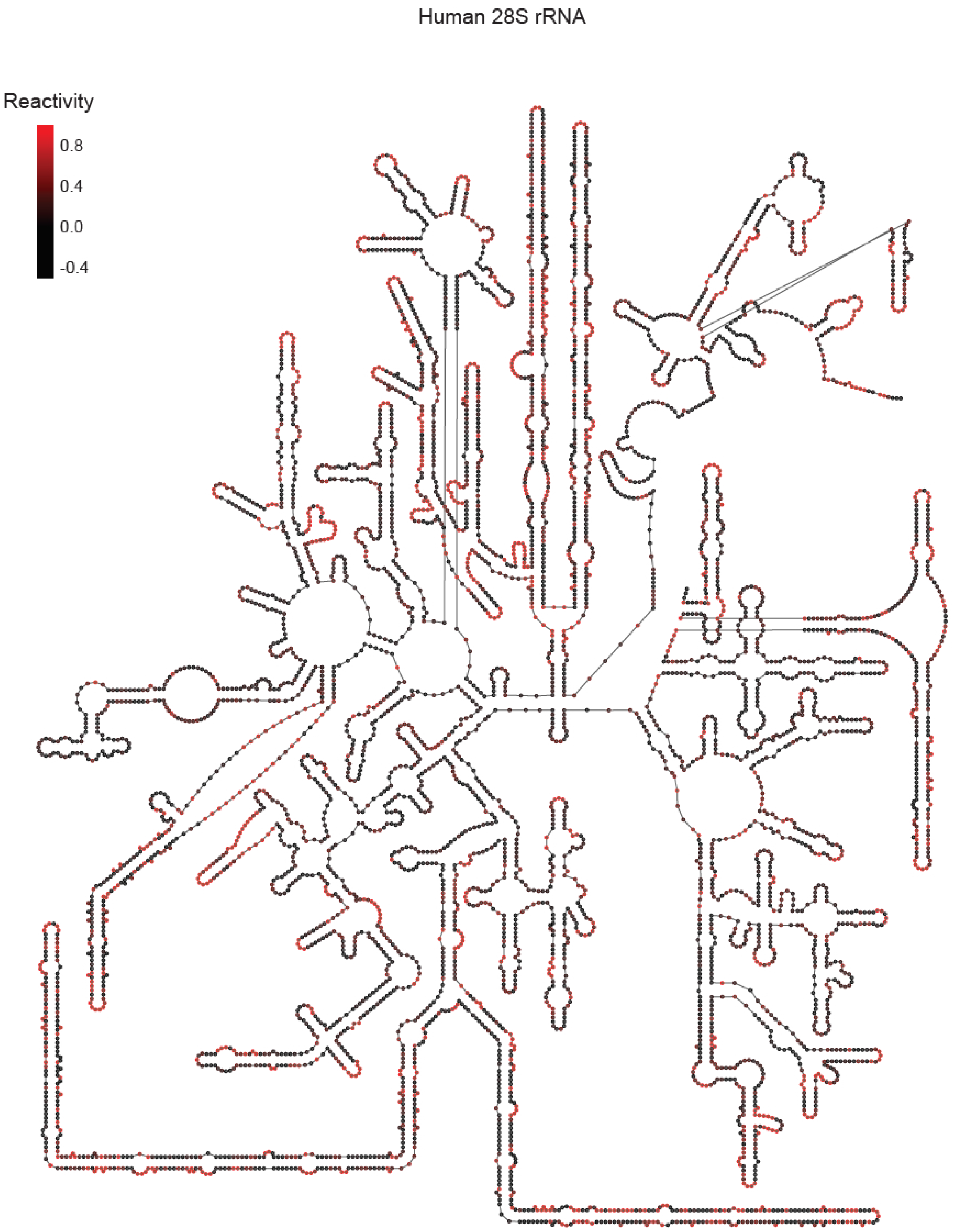

Supplementary Figure 1. NAI-MaP identifies single stranded regions accurately in vivo. NAI-MaP reactivities on the 28S rRNA in Hela cells. Highly reactive NAI-MaP signals (red) map to known single-stranded regions along the 28S rRNA secondary structure.

Supplementary Figure 2

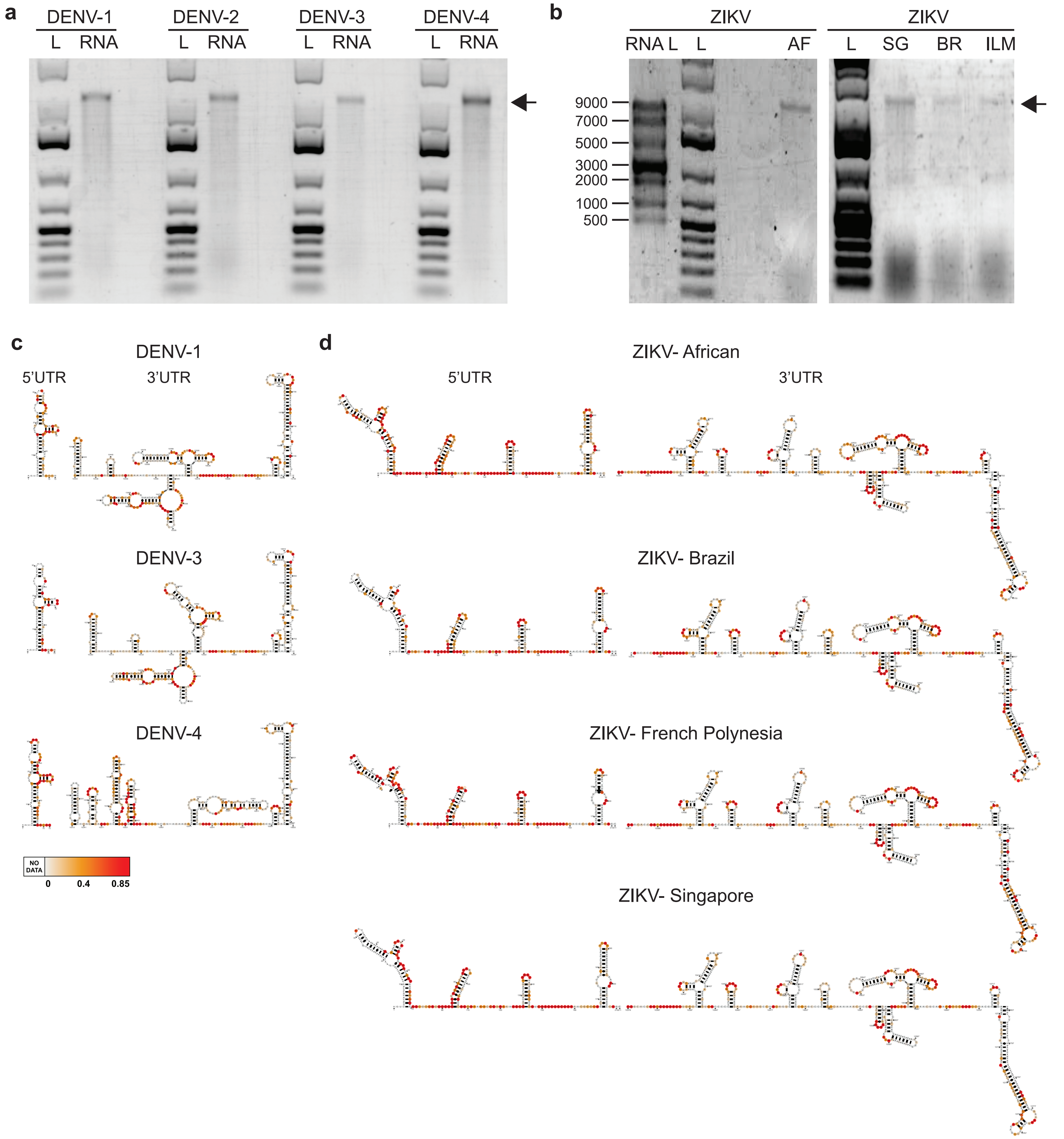

Supplementary Figure 2. Visualization of NAI-MaP reactivities of DENV and ZIKA viruses on known reference structures. a,b, Gel images showing that structure probing was performed on full length, intact, DENV (A) and ZIKV (B) genomes. Extracted RNA is run on 0.6% agarose gels using DNA and RNA ladders as reference. L is 1kb plus DNA ladder (GeneRuler), RNA L is ssRNA ladder (NEB). **c, d,** NAI-MaP reactivities mapped to 5' and 3'UTR structure models in DENV (**c**) and ZIKV (**d**) genomes. High reactivities are indicated in red, and suggests increased likelihood of a base being single-stranded.

Supplementary Figure 3

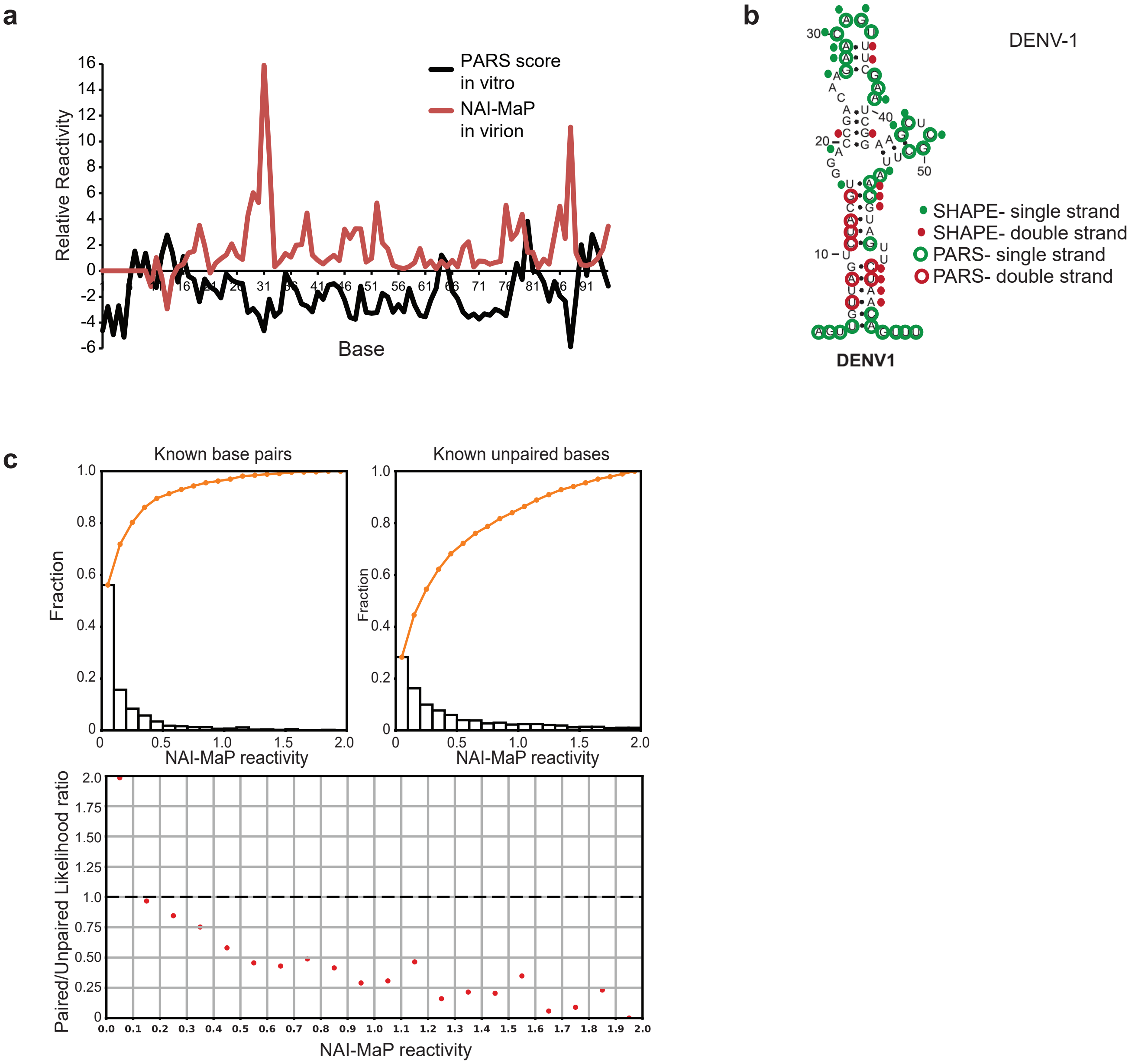

Supplementary Figure 3. Properties of NAI-MaP reactivities in DENV and ZIKA genomes. a, In vitro structure probing of the DENV-1 genome using enzymatic digestion followed by deep sequencing (PARS, red line), or NAI-MaP (black line). High reactivity in NAI-MaP indicates that a base is single-stranded while high signal for PARS indicates that a base is double stranded. PARS and NAI-MaP signals are largely inverse of each other, suggesting that they are identifying double and single stranded bases at similar positions. **b**, PARS and NAI-MaP are mapped to the known 5'UTR structure in DENV-1. **c**, Top, distribution of NAI-MaP reactivities from our data sets in bases that are known to be paired (left) and unpaired (right) in previously described 5' and 3'UTR structures of DENV and ZIKV. Unpaired bases have a greater fraction of bases with high NAI-MaP reactivity, although the mode of the distribution is also at 0. Bottom: A plot of the likelihood ratio of a base being paired or unpaired versus its NAI-MaP reactivity. The higher the NAI-MaP reactivity, the more likely a base is to be single-stranded.

Supplementary Figure 4

a

Sequence identities between different serotypes

	DENV-1	DENV-2	DENV-3	DENV-4	ZIKA-AFR	ZIKA-BRA	ZIKA-FP	ZIKA-SIN
**DENV-1**	100	68.63	72.79	67.06	58.86	58.53	58.58	58.53
**DENV-2**	68.63	100	69.07	67.51	59.19	58.58	58.6	58.57
**DENV-3**	72.79	69.07	100	67.37	58.82	58.56	58.56	58.55
**DENV-4**	67.06	67.51	67.37	100	58.51	58.54	58.65	58.62
**ZIKA-AFR**	58.86	59.19	58.82	58.51	100	88.81	88.94	88.96
**ZIKA-BRA**	58.53	58.58	58.56	58.54	88.81	100	99.65	99.17
**ZIKA-FP**	58.58	58.6	58.56	58.65	88.94	99.65	100	99.4
**ZIKA-SIN**	58.53	58.57	58.55	58.62	88.96	99.17	99.4	100

b

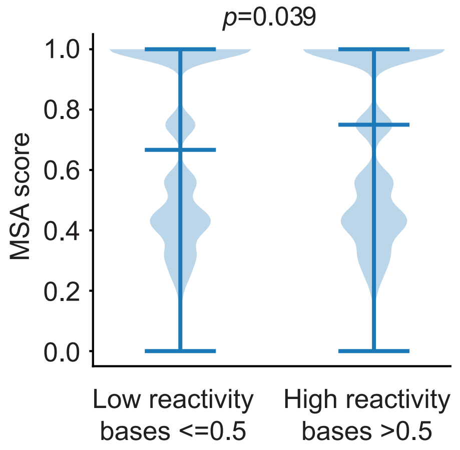

d

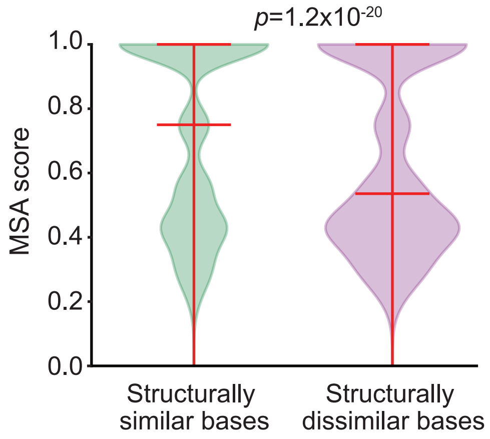

Supplementary Figure 4. Sequence and structure features of DENV and ZIKV. **a**, Sequence identities between DENV and ZIKV strains. **b**, Violin plots showing the distribution of sequence similarity across eight viruses in positions that have low reactivity (more structured), versus positions that have high reactivity (less structured). Single stranded regions tend to be more highly sequence conserved across the eight viruses as compared to double stranded regions. **c**, Violin plots showing the distribution of sequence similarity across the eight DENV and ZIKV viruses in positions that are structurally similar (left) and structurally different (right) across the viruses. Bases that share similarities in structure tend to share similarity in sequence. **b,c**, The lines on the violin plots indicate median, minimum and maximum values of the distribution. *P*-values are calculated by Wilcoxon Ranked Sum Test.

Supplementary Figure 5

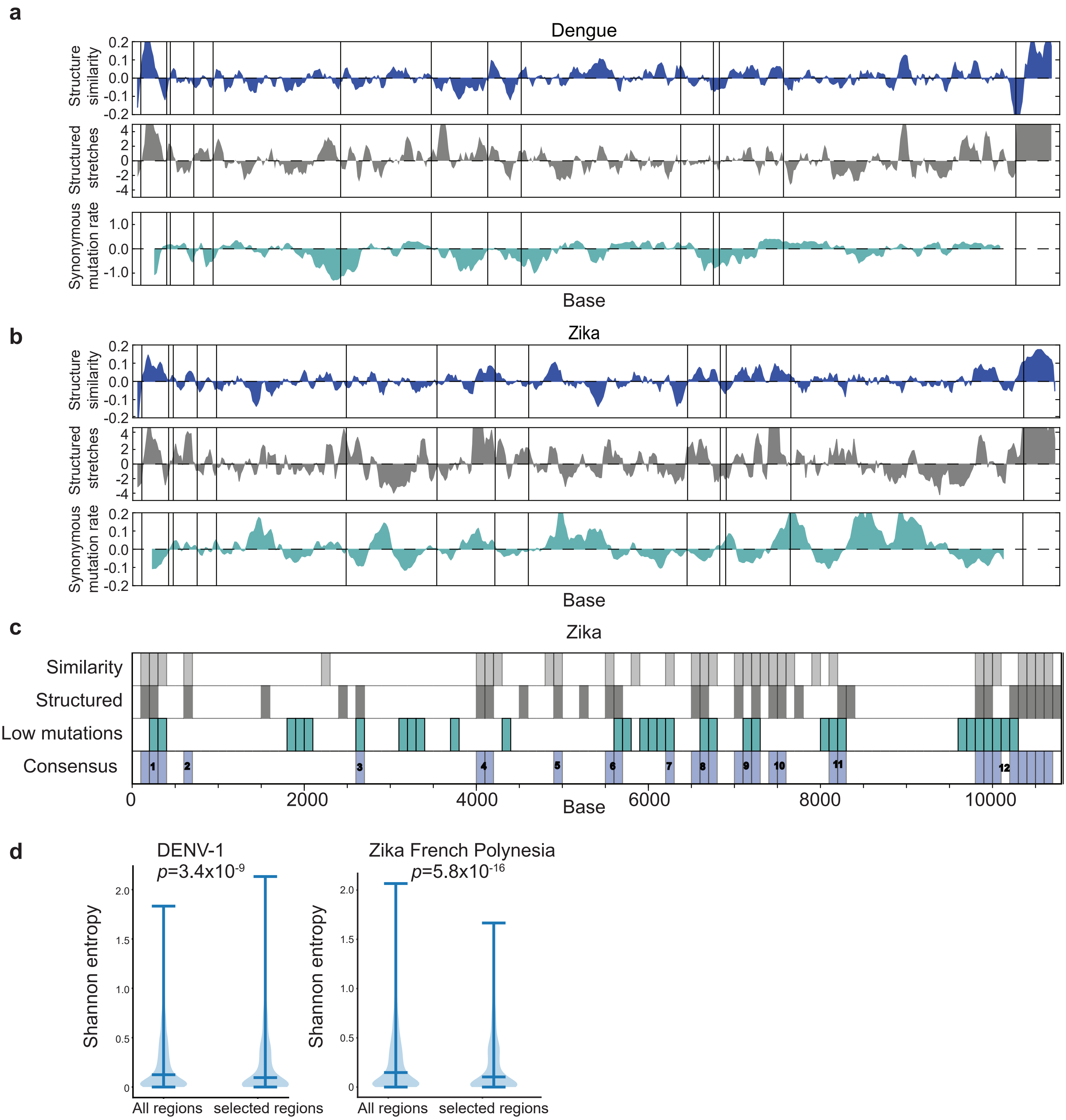

Supplementary Figure 5. Analysis of conserved local structural regions in DENV and ZIKV. a, b, Plots showing the extent of structural similarity, double-strandedness (structured stretches), and the extent of synonymous mutation rate across the four DENV (a) and four ZIKV (b) genomes. Structure similarity is calculated using pearson correlation of 100 base windows along the genome. Structured structures is the inverse of NAI-MaP reactivities (The lower the NAI-MaP reactivity in the 100 base window, the more structured that window is). The top 30 percentile of each of these plots are shown as blocks in Figure 2a, and Supplementary Figure 4c. **c**, Plots show 100 nucleotide regions across four DENV genomes that have highly similar structures (grey), are highly double-stranded (black), and accumulate low levels of synonymous mutations (blue). Regions that fulfil two out of three of the above criteria are selected as consensus regions (purple) and are potentially functionally important. **d**, Violin plots showing the distribution of Shannon entropies in the conserved 16 DENV (left) versus 12 ZIKV (right) structures (selected regions) versus all regions along the genome. The conserved structures show lower Shannon entropies, suggesting that they form unique structures. The lines indicate maximum, median and minimum values in the violin plot. *P*-value was calculated by the Wilcoxon Rank Sum test.

Supplementary Figure 6

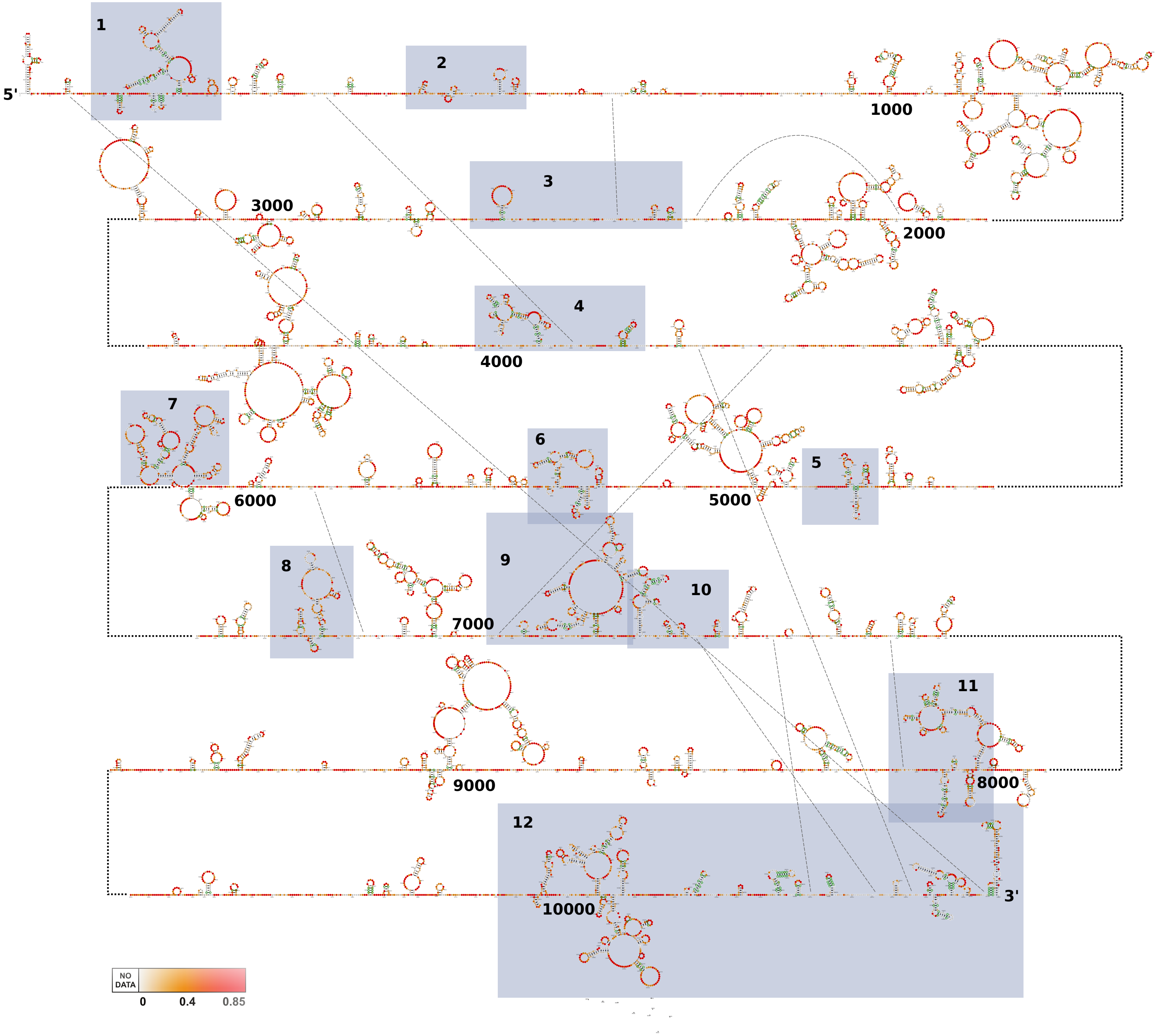

Supplementary Figure 6. Structure model of the ZIKV French Polynesia genome using NAI-MaP as experimental constraints. The grey boxes indicates the 12 conserved RNA regions identified in ZIKV. Bases with high reactivity are indicated in red and bases with evidence of covariation are circled in green. Out of a 1000 possible NAI-MaP constrained structures, this structure is the most concordant with known 5' and 3'UTR structures in the literature.

Supplementary Figure 7

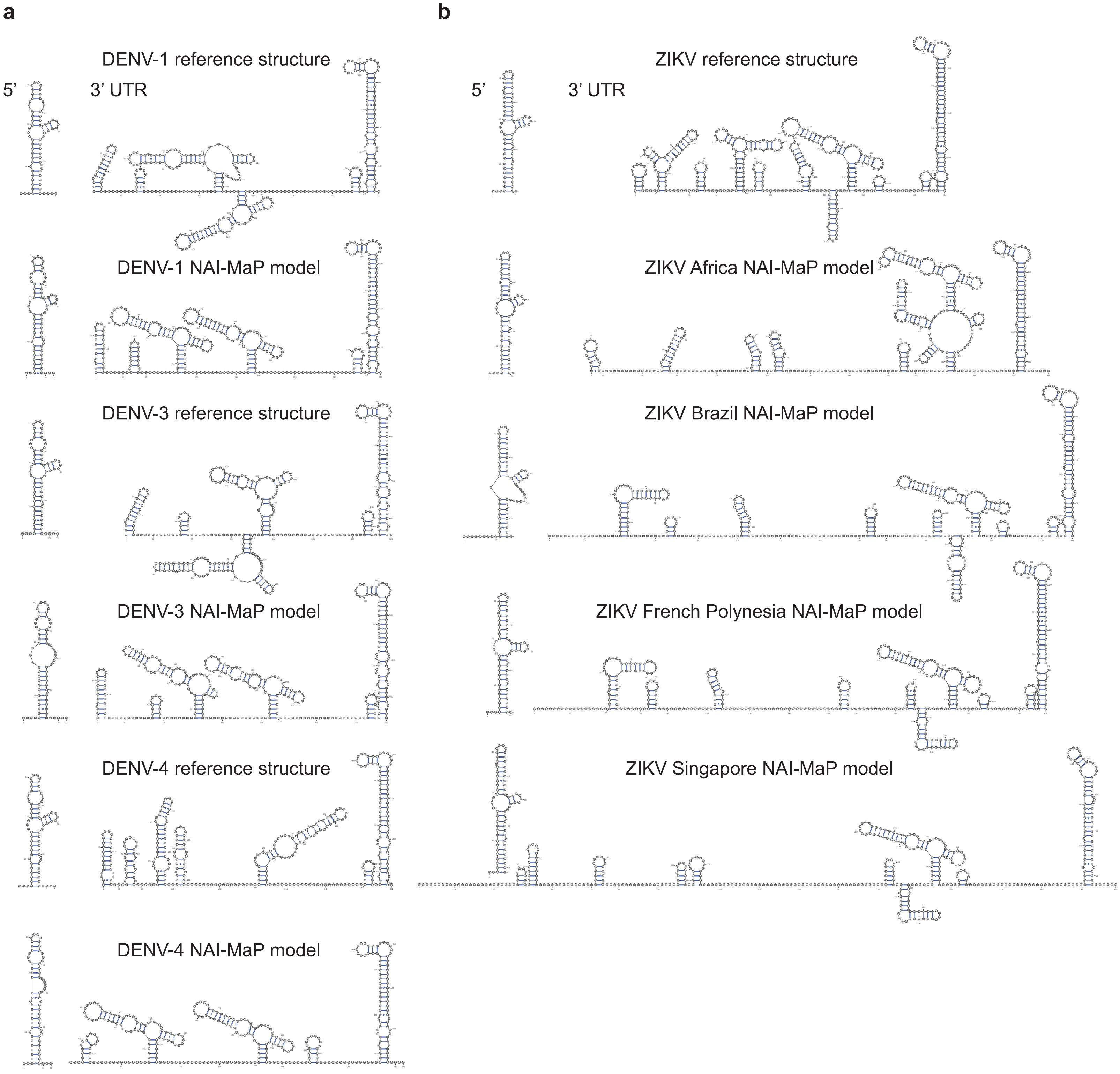

Supplementary Figure 7. Reference and NAI-MaP constrained DENV and ZIKV structure models. Dengue (**a**) and Zika (**b**) NAI-MaP constrained 5' and 3'UTRs secondary structure models.

Supplementary Figure 8

a

% correct base pairs		
	-NAI-shape	+NAI-shape
16S	41.36	84.25
23S	58.67	79.4

b

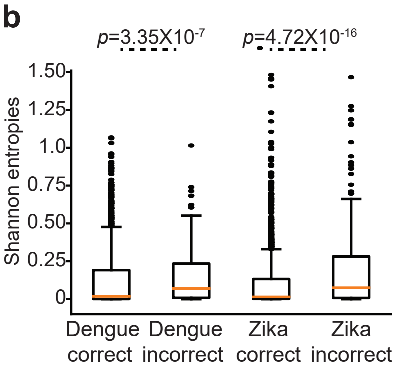

c

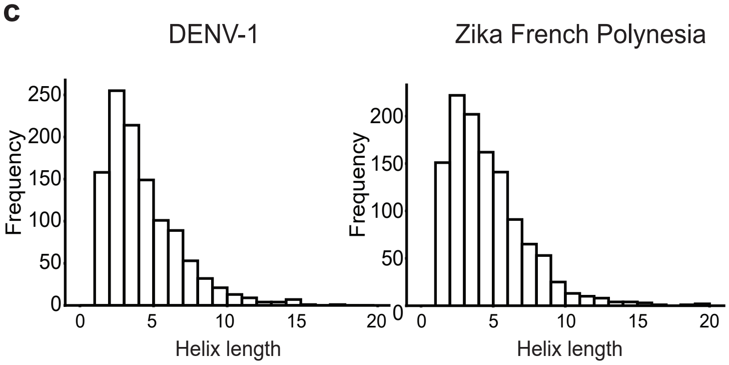

d

	DENV-1	DENV-2	DENV-3	DENV-4	ZAFR	ZBRA	ZFP	ZSIN
Median length	4	4	4	4	4	4	4	4
% <= 7 bases	90.3	89.8	89.2	89.3	88.5	88.5	87.7	85.6
% > 9 bases	9.6	10.2	10.8	10.7	11.5	11.5	12.3	14.4
Longest helix length	17	16	16	17	20	20	19	17

Supplementary Figure 8. Statistics of NAI-MaP constrained structure models. a, Table showing the percentage of correct base pairs that are modelled in 16S and 23S rRNA with and without incorporating NAI-MaP reactivities into the RNAstructure program. **b**, Boxplot showing the distribution of Shannon entropies in bases with reactivities that agree with the modelled DENV and ZIKV structures, versus bases with reactivities that do not agree with the modelled structures. **c**, Histogram of the distribution of the length of stems that are present in modelled DENV and ZIKV RNA structures after incorporating NAI-MaP reactivities into RNA structure program. **d**, Table containing information on the length of helices in DENV and ZIKV serotypes.

Supplementary Figure 9

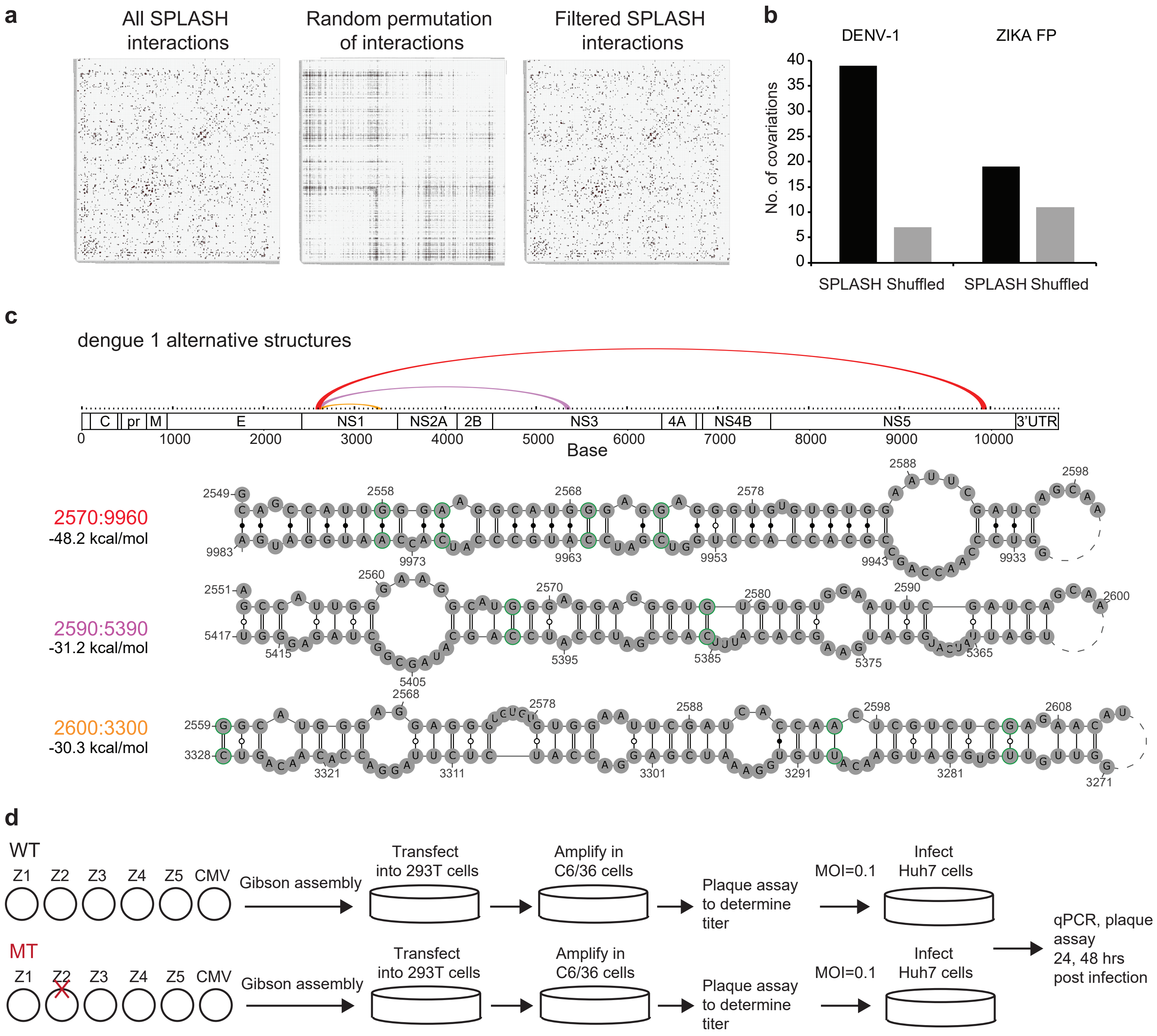

Supplementary Figure 9. Many long-range interactions are identified in DENV and ZIKV viruses. a, 2D matrices showing the location of pair-wise RNA interactions along the DENV1 genome inside virions (left), upon random shuffling (middle), and upon filtering against random shuffled interactions (right). **b**, Number of covaried bases in top 100 SPLASH interactions versus shuffled interactions in DENV-1 and ZIKV French Polynesia. SPLASH interactions are enriched for covaried bases, supporting the importance of these interactions through evolution. **c**, Top: arc plots showing one set of alternative interactions found along the ZIKV French Polynesia genome. Bottom: structure models of the pair-wise interactions for the alternative structures, using the program RNAcofold. The NAI-MaP reactivities (red, orange, black, indicating single, intermediate, and double stranded regions respectively) and covariation information (green circles) are mapped onto the structures. **d**, Schematic of the functional assay to test for mutant virus fitness (see Methods).

Supplementary Figure 10

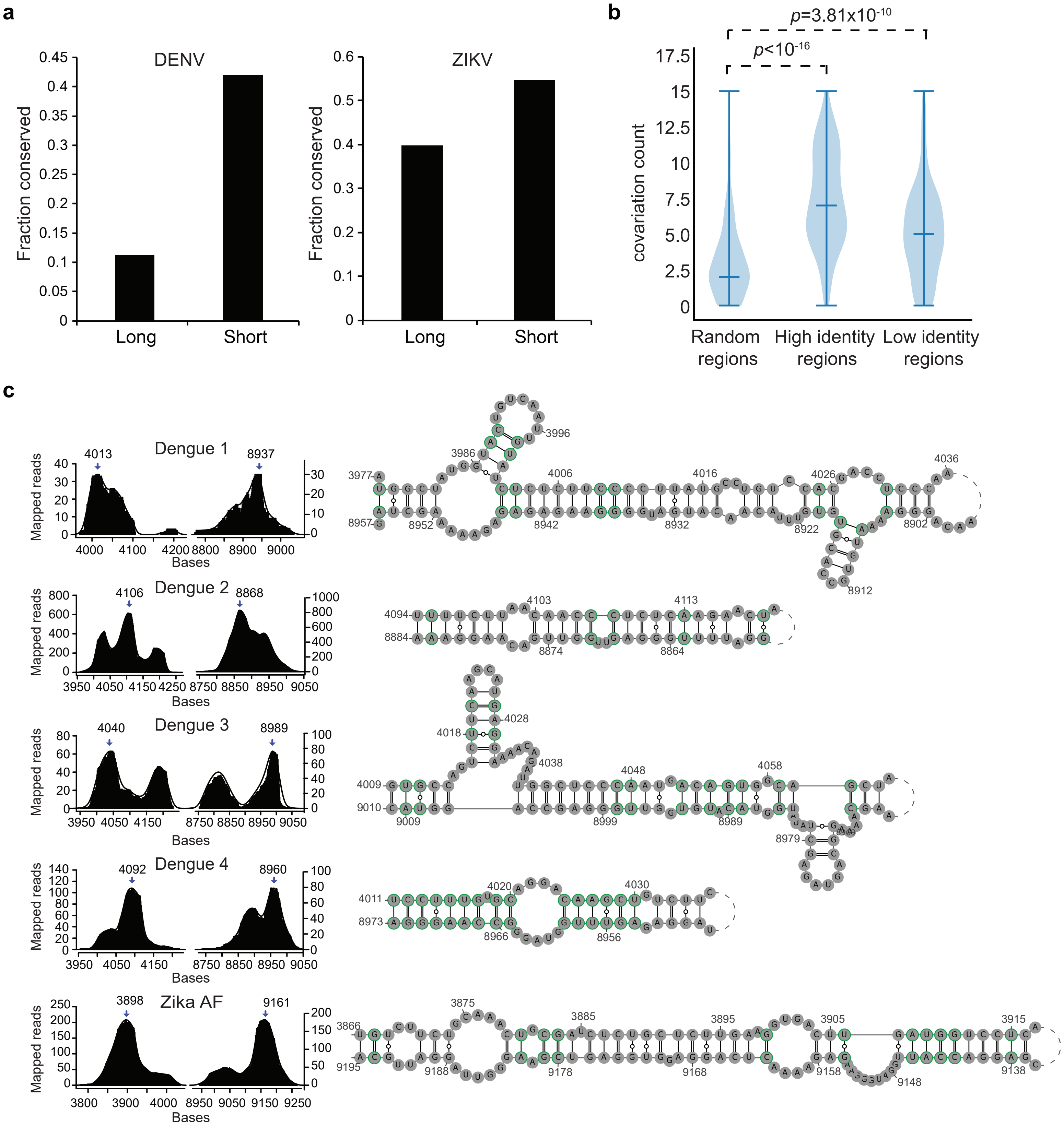

Supplementary Figure 10. Features of conserved pair-wise interactions in DENV and ZIKV viruses. a, Fraction of long (>=500 bases) and short (<500 bases) pair-wise interactions that are conserved in two or more DENV and ZIKV. **b**, Violin plots showing the distribution of average local covariation at locations selected from random in the genome (left), SPLASH interactions with higher than average sequence identity (middle), and with lower than average sequence identity (Right) across the 8 virus serotypes. **c**, Structure models of the predicted interactions between bases 4000:9000 in the different DENV and ZIKV viruses are generated using the program RNAcofold. NAI-MaP reactivities (red, orange, black, indicating single, intermediate, and double stranded regions respectively) and covariation information (green circles) are mapped onto the structures.

Supplementary Figure 11

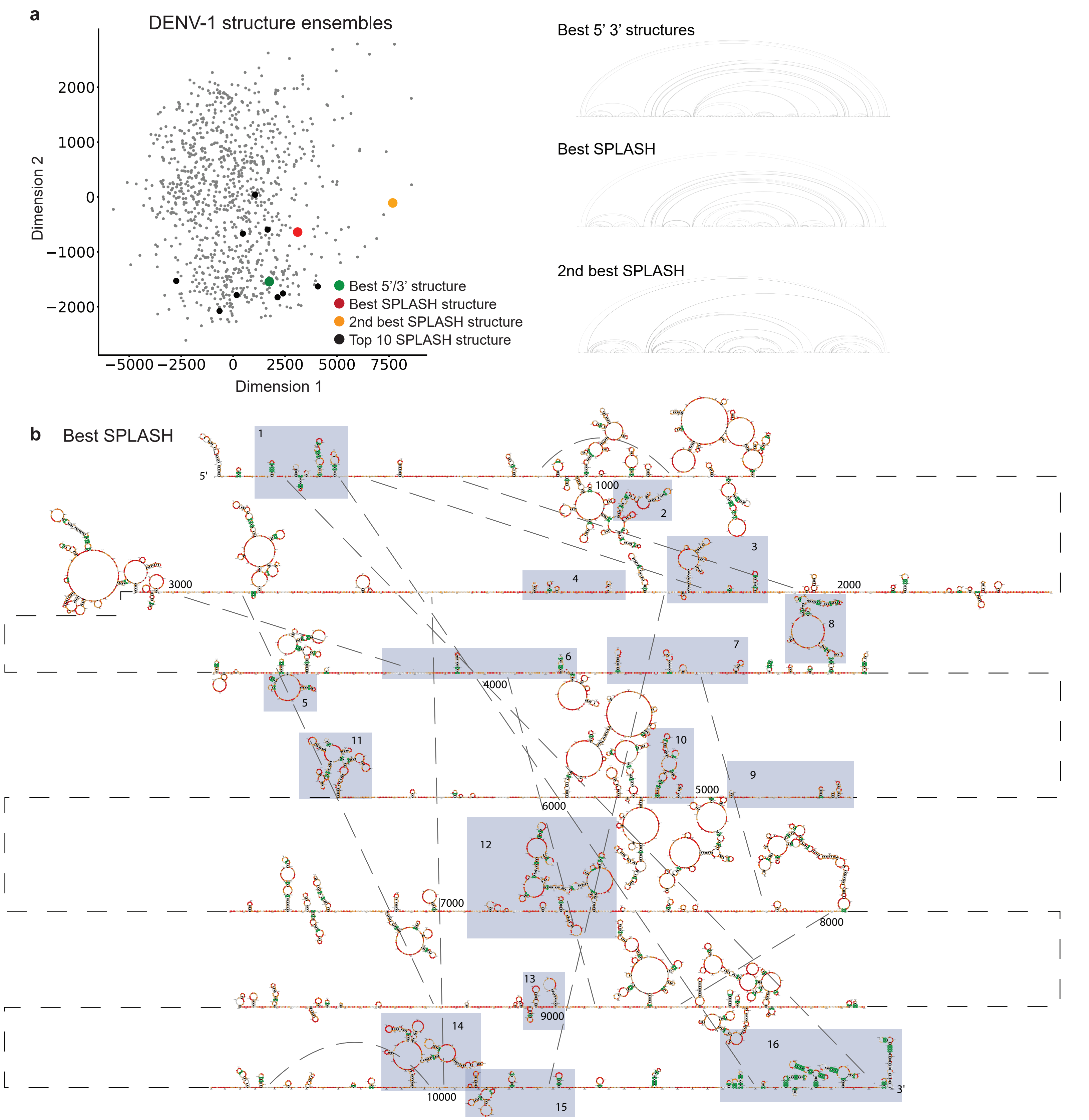

Supplementary Figure 11. Combining NAI-MaP and SPLASH information for DENV structure modelling. a, NAI-MaP reactivities were first used as structural constraints to generate an ensemble of 1000 potential structures. Structures that best fit SPLASH interactions for DENV were then selected from the structure ensembles. Left: Clustering of 1000 NAI-MaP constrained structures. The red and yellow dots represents the structure with the largest and second largest concordance with SPLASH interactions. The black dots represent the structures with the top 10 best concordance with SPLASH. Also highlighted is the structure with the most accurate 5' and 3'UTRs based on known DENV structures (Green dot). Right: Arc plots showing the pair-wise interactions in each structure selected based on NAI-MaP constraints and best 5'/3' UTR correctness or with largest or second largest concordance with SPLASH. b, Structure model of DENV-1 constrained using NAI-MaP reactivity and most concordant with SPLASH interactions. The grey boxes indicate the 16 conserved RNA regions in DENV. Bases with high reactivity are indicated as red and bases that are covaried are in green.

Supplementary Figure 12

Second best fit with SPLASH

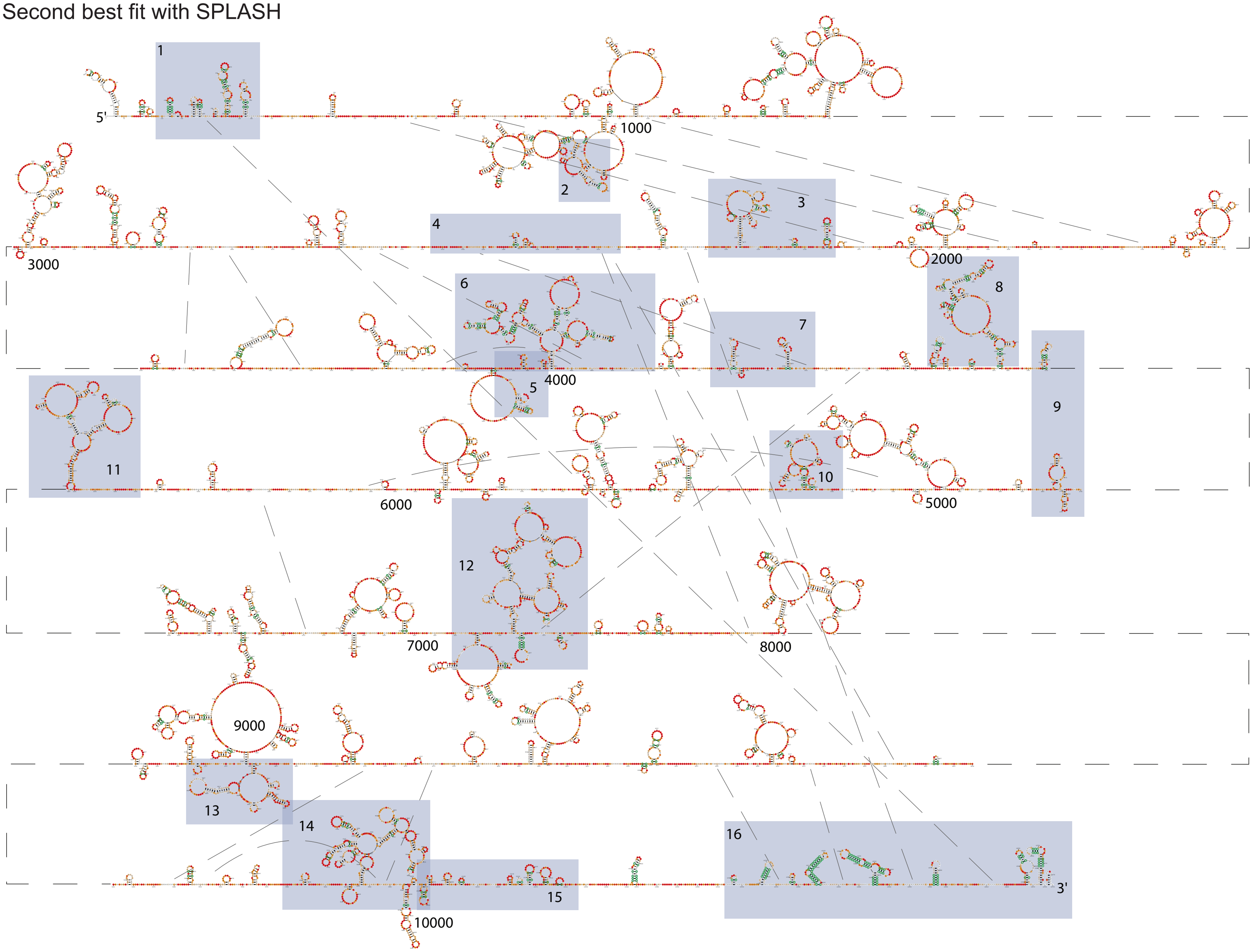

Supplementary Figure 12. Structure model of DENV-1 constrained using NAI-MaP reactivity and with the second highest concordance with SPLASH interactions. The grey boxes indicate the 16 conserved RNA regions in DENV. Bases with high reactivity are indicated as red and bases that are covaried are in green.

Supplementary Figure 13

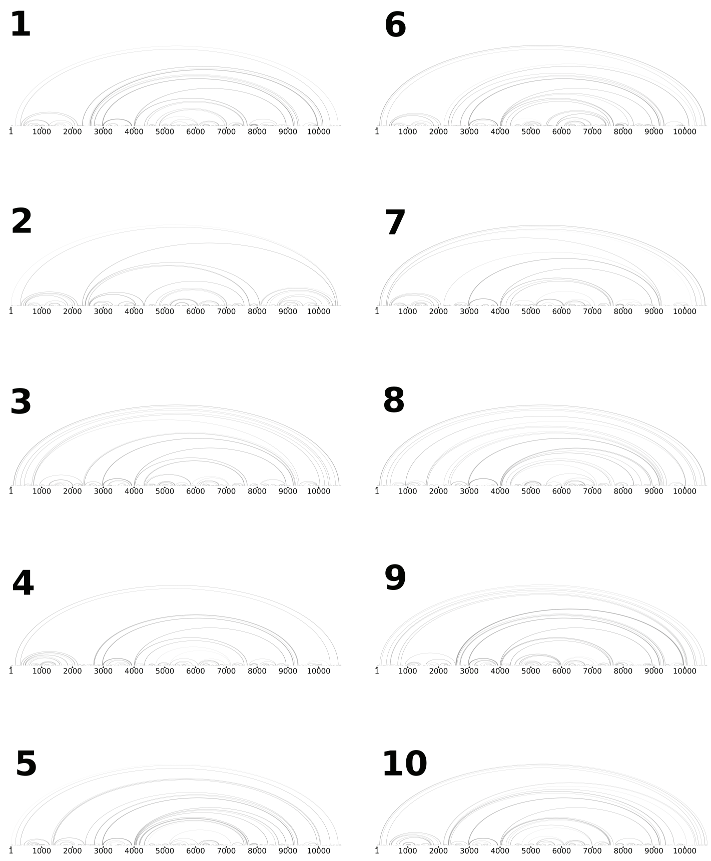

Supplementary Figure 13. Heterogeneity in genome organization inside dengue virions. Arc plots showing the pair-wise interactions in each structure selected based on NAI-MaP constraints and the top 10 largest concordance with SPLASH.

Supplementary Figure 14

a

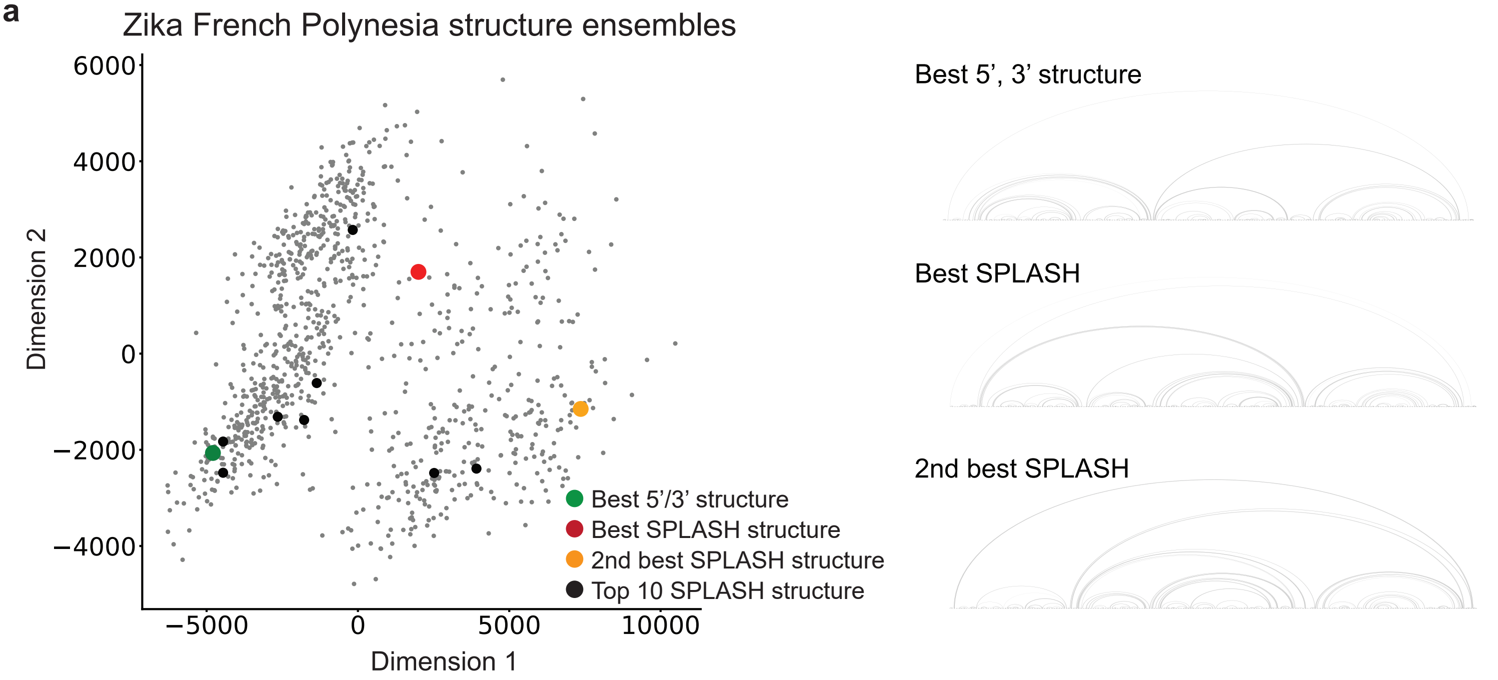

b

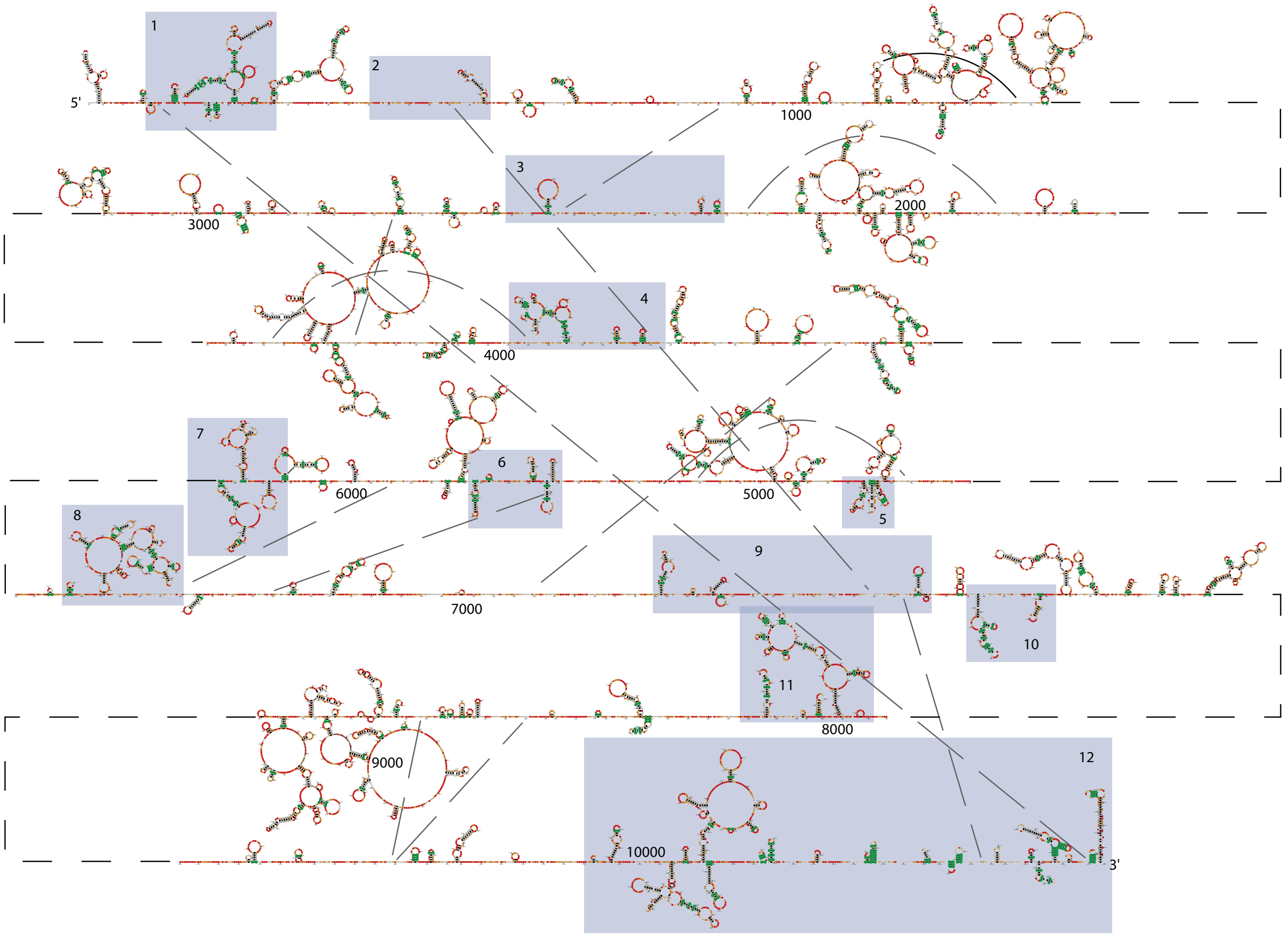

Supplementary Figure 14. Combining NAI-MaP and SPLASH information for ZIKV structure modelling. a, NAI-MaP reactivities were first used as structural constraints to generate an ensemble of 1000 potential structures. Structures that best fit SPLASH interactions for ZIKV were then selected from the structure ensembles. Left: Clustering of 1000 NAI-MaP constrained structures. The red and yellow dots represents the structure with the largest and second largest concordance with SPLASH interactions. The black dots represent the structures with the top 10 best concordance with SPLASH. Also highlighted is the structure with the most accurate 5' and 3'UTRs based on known ZIKV structures (Green dot). Right: Arc plots showing the pair-wise interactions in each structure selected based on NAI-MaP constraints and best 5'/3' UTR correctness or with largest or second largest concordance with SPLASH. b, Structure model of ZIKV genome that is NAI-MaP constrained, and has the most concordance with SPLASH data. The grey boxes indicated conserved regions in ZIKV.

Supplementary Figure 15

Second best fit with SPLASH

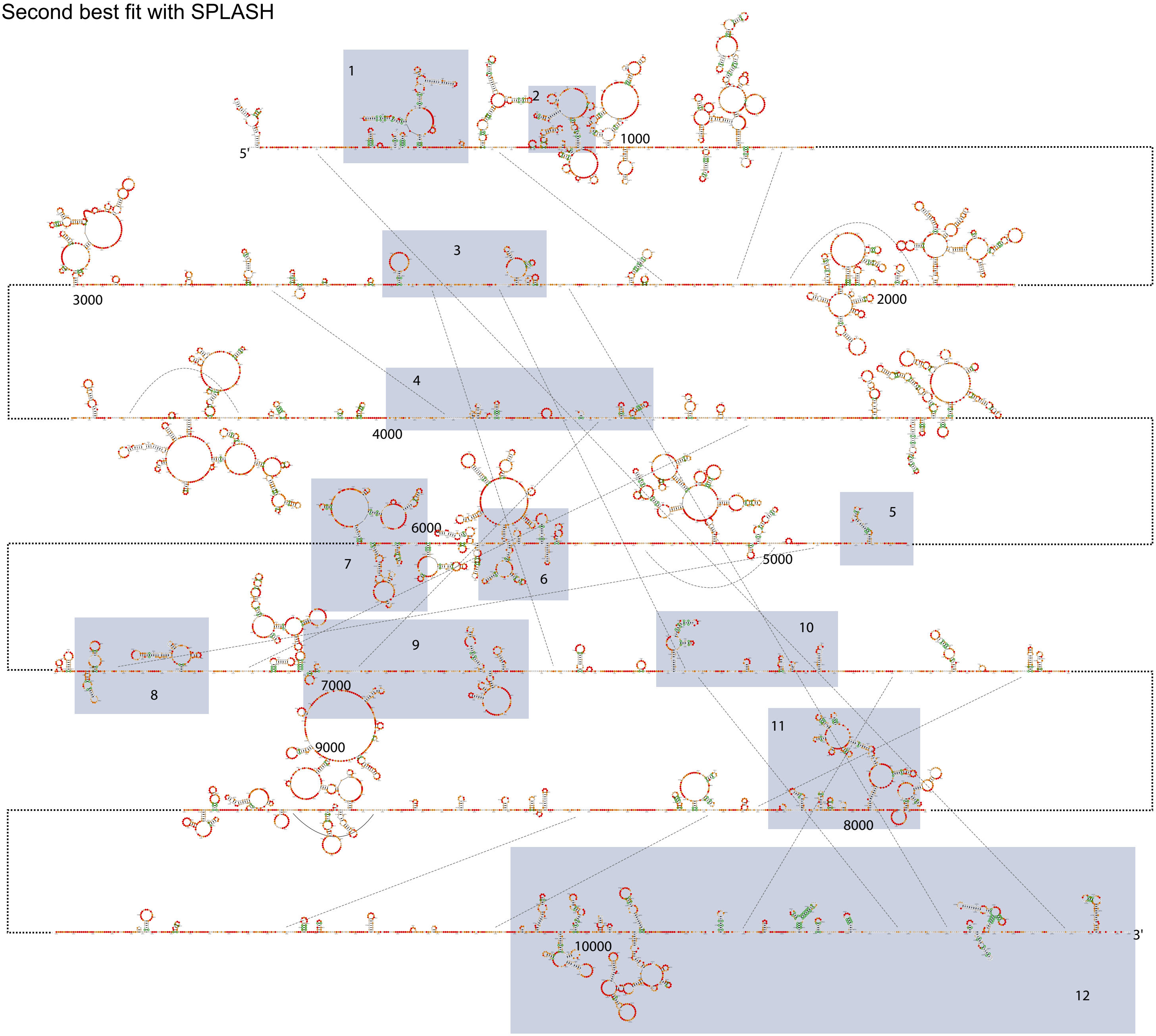

Supplementary Figure 15. Structure model of ZIKV French Polynesia constrained using NAI-MaP reactivity and selected by second highest concordance with SPLASH. The grey boxes indicated the 12 conserved RNA regions in ZIKV. Bases with high NAI-MaP reactivity are indicated in red, and co-varied bases are indicated in green.

Supplementary Figure 16

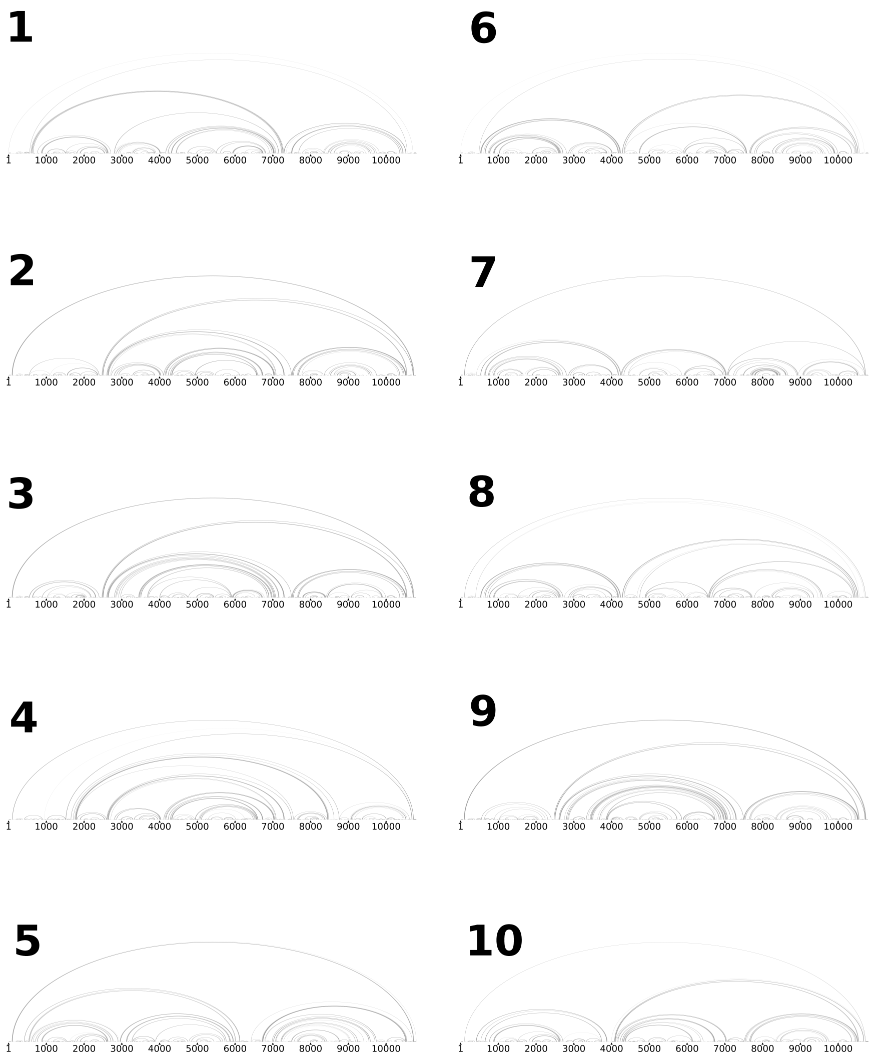

Supplementary Figure 16. Heterogeneity in genome organization inside Zika virions. Arc plots showing the pair-wise interactions in each structure selected based on NAI-MaP constraints and the top 10 largest concordance with SPLASH.

Supplementary Figure 17

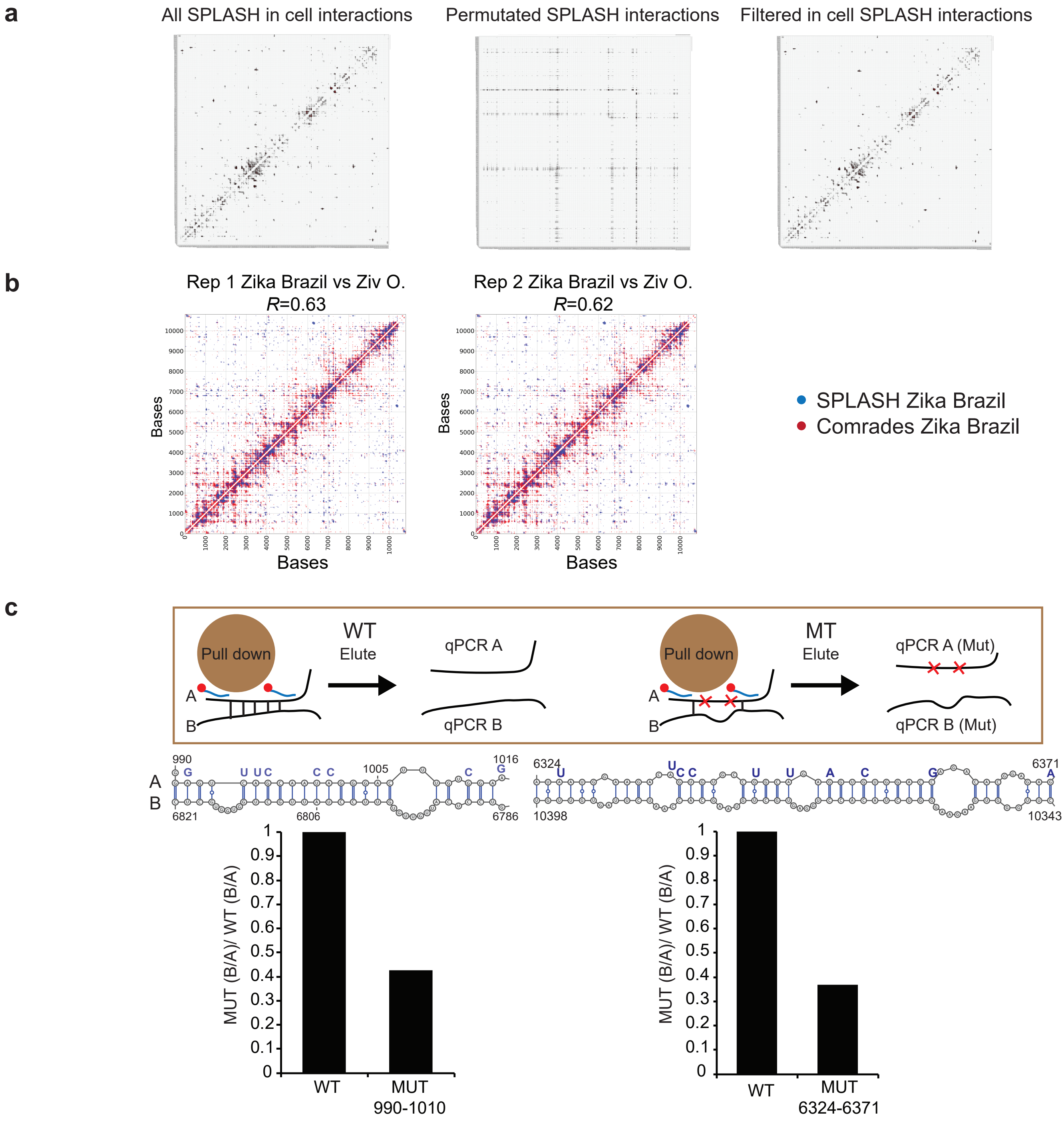

Supplementary Figure 17. Validation of SPLASH interactions in DENV and ZIKV. a, 2D matrices showing the location of pair-wise RNA interactions along the DENV1 genome inside cells (left), upon random shuffling (middle), and upon filtering against random shuffled interactions (right). In cell pair-wise interactions are enriched for short, local interactions along the diagonal of the plot. **b**, 2-dimensional matrices indicating the position of pair-wise RNA interactions in ZIKV Brazil strain, as identified by SPLASH (in blue) and by COMRADES (in red). SPLASH data shows good correlation with COMRADES data (Pearson, *R*=0.62, *R*=0.63 for 2 independent replicates). **c**, Validation of pair-wise RNA interactions inside cells by mutational and pulldown experiments. Top: Biotinylated probes against one strand of the pair-wise interaction was used to pull down the wildtype and the mutated interaction using streptavidin beads. qPCR analysis was performed on the other complementary strand to determine the amount of complementary binding. If the pair-wise interactions exist, mutations that disrupt the pairing will result in a lower amount of the complementary strand to be pulled down as compared to wildtype interactions. Bottom: Schematic showing the mutations that were made on the pair-wise RNA interactions. Plot of the amount of complementary strand that was detected in wildtype and in mutated interactions. Mutations along pair-wise RNA interactions result in less complementary strand being detected, confirming that the mutations indeed disrupt the interaction.

Supplementary Figure 18

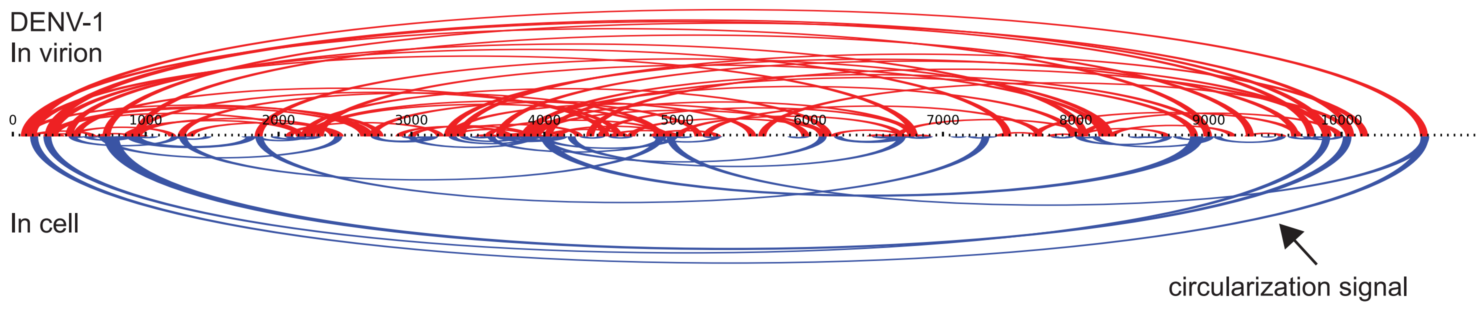

Supplementary Figure 18, In cell and in virion interactions in DENV-1 virus. Arc plots showing long-range interactions in DENV-1 virus that are present inside virion particles (top) versus inside host cells (bottom).

Supplementary Table 1. Statistics of NAI-MaP and SPLASH sequencing libraries

	**DENV-1**	**DENV-2**	**DENV-3**	**DENV- 4**	**ZIKV- African**	**ZIKV- Brazil**	**ZIKV- French Polynesia**	**ZIKV- Singapore**
**NCBI Accession ID**	EU081230	EU081177	EU081190	GQ398256	AY632535	KU497555	KJ776791	KY241787
**Genome Length**	10735 bp	10723 bp	10706 bp	10653 bp	10794 bp	10793 bp	10807 bp	10770 bp
**NAI-MaP N(reads)**	177M	184M	107M	188M	123M	150M	134M	125M
**NAI-MaP Coverage (99th percentile)**	6426	5418	4588	9036	7039	13414	10681	15168
**NAI-MaP Correlation**	0.9	0.86	0.98	0.93	0.81	0.84	0.88	0.85
**SPLASH N(reads) VIRION**	9.4M	80M	41M	53M	57M	178M	63M	253M
**SPLASH Chimeric Reads VIRION**	13k	284k	107k	115k	115k	260k	311k	220k
**SPLASH N(reads) CELL**	191M	79M	110M	104M	194M	188M	157M	153M
**SPLASH Chimeric Reads CELL**	101k	0.2k	13k	5k	39k	19k	9k	17k
