# Supplementary material for: Structure mapping of dengue and Zika viruses reveals functional long-range interactions
Source: Nat Commun. 2019 Mar 29;10:1408. doi: 10.1038/s41467-019-09391-8 (PMC6441010; doi:10.1038/s41467-019-09391-8)
Supplement: Supplementary file 4 — Description of Additional Supplementary Files [file 41467_2019_9391_MOESM4_ESM.pdf]

Description of Additional Supplementary Files

Supplementary Data 1. NAI-MaP reactivities for 8 viruses inside virions. Column 1 is the position of the reference alignment, Columns 2-9 contains the positions along each virus according to the reference alignment. Columns 10-17 contains the individual NAI-MaP reactivities for each base for each virus.

Supplementary Data 2. SPLASH interactions in DENV1-4 inside virions. For each virus: Columns 1 and 2 contain the left and right nucleotide positions of an individual pair-wise interaction respectively. Columns 3 and 4 contain the left and right nucleotide sequences of each pair-wise interaction respectively. Column 5 contains information on the number of times the interaction has been observed in our experiments.

Supplementary Data 3. SPLASH interactions in the four Zika viruses inside virions. For each virus: Columns 1 and 2 contain the left and right nucleotide positions of an individual pair-wise interaction respectively. Columns 3 and 4 contain the left and right nucleotide sequences of each pair-wise interaction respectively. Column 5 contains information on the number of times the interaction has been observed in our experiments.

Supplementary Data 4. Consensus DENV SPLASH interactions that are shared either inside infected cells or inside virions. Both inside cells, and inside virions: Columns 1 and 2 contain nucleotide positions of the shared interaction, and Column 3 contains information on which strains the interaction is found in.

Supplementary Data 5. Consensus ZIKV SPLASH interactions that are shared either inside infected cells or inside virions. Both inside cells, and inside virions: Columns 1 and 2 contain nucleotide positions of the shared interaction, and Column 3 contains information on which strains the interaction is found in.

Supplementary Data 6. Alignment of dengue and Zika sequences as input for R-Scape to identify co-variation information in DENV and ZIKV.

Supplementary Data 7. R-Scape output for the DENV and ZIKV structure models. Columns contain the pair-wise positions, score, E-value and number of substitutions of each co-varied base for the DENV and ZIKV structure with 1) best fit to known UTR structures in the literature, 2) best fit to SPLASH data and 3) second best fit to SPLASH data.

Supplementary Data 8. SPLASH interactions in DENV1-4 inside cells. For each virus: Columns 1 and 2 contain the left and right nucleotide positions of an individual pair-wise interaction respectively. Columns 3 and 4 contain the left and right nucleotide sequences of each pair-wise interaction respectively. Column 5 contains information on the number of times the interaction has been observed in our experiments.

Supplementary Data 9. SPLASH interactions in the four Zika viruses inside cells. For each virus: Columns 1 and 2 contain the left and right nucleotide positions of an individual pair-wise interaction respectively. Columns 3 and 4 contain the left and right nucleotide sequences of each pair-wise interaction respectively. Column 5 contains information on the number of times the interaction has been observed in our experiments.
